# Father

**Published:** 1915-01

**Authors:** Ella Wheeler Wilcox


### The Department of Eugenics, Mental and Social Hygiene

CONDUCTED BY
DR. MALONE DUGGAN
813-814 Gibbs Building, San Antonio, Texas

All communications for this department should be sent to DR. DUGGAN

### Father.

BY ELLA WHEELER WILCOX.

He had never made a fortune or a noise,
In the world where men are seeking after fame;
But he had a healthy brood of girls and boys,
Who loved the very ground on which he trod.
They thought him a little short of God;
Oh, you should have heard the way they said his name—
"Father."

There seemed to be a little tender prayer
In their voices even when they called him dad,
Though the man was never heard of anywhere
As a hero, yet you somehow understood
He was doing well his part and making good,
And you knew it by the way the children had of saying—
"Father."

He gave them neither eminence nor wealth,
But he gave them blood untainted with a vice;
And the opulence of undiluted health.
He was honest and unpurchasable and kind.
He was clean in heart and body, and in mind,
So he made them heirs to riches beyond price—
This Father.

—*Exchange.*

